# Evolution of coordinated punishment to enforce cooperation from an unbiased strategy space

**DOI:** 10.1098/rsif.2019.0127

**Published:** 2019-07-24

**Authors:** Julián García, Arne Traulsen

**Affiliations:** 1 Faculty of Information Technology, Monash University, Melbourne, Australia; 2 Department of Evolutionary Theory, Max Planck Institute for Evolutionary Biology, 24306 Plön, Germany

**Keywords:** collective action, punishment, evolution, cooperation

## Abstract

The emergence and maintenance of punishment to protect the commons remains an open puzzle in social and biological sciences. Even in societies where pro-social punishing is common, some individuals seek to cheat the system if they see a chance to do so—and public goods are often maintained in spite of cheaters who do not contribute. We present a model accounting for all possible strategies in a public goods game with punishment. While most models of punishment restrict the set of possible behaviours, excluding seemingly paradoxical anti-social strategies from the start, we show that these strategies can play an important role in explaining large-scale cooperation as observed in human societies. We find that coordinated punishment can emerge from individual interactions, but the stability of the associated institutions is limited owing to anti-social and opportunistic behaviour. In particular, coordinated anti-social punishment can undermine cooperation if individuals cannot condition their behaviour on the existence of institutions that punish. Only when we allow for observability and conditional behaviours do anti-social strategies no longer threaten cooperation. This is due to a stable coexistence of a minority supporting pro-social institutions and those who only cooperate if such institutions are in place. This minority of supporters is enough to guarantee substantial cooperation under a wide range of conditions. Our findings resonate with the empirical observation that public goods are resilient to opportunistic cheaters in large groups of unrelated individuals. They also highlight the importance of letting evolution, and not modellers, decide which strategies matter.

## 1. Introduction

Most modern societies have put in place institutions that support and promote collective action. Understanding the origin of these institutions is an important challenge across biological and social sciences [1]. The outstanding capacity of humans to engage in large-scale cooperation often relies on these institutionalized enforcement mechanisms [2]. Centralized institutions for cooperation also have experimental and empirical support [3,4], but explaining how these institutions arise from individual incentives is an open problem [5]. Here, we propose that these institutions play a role in enabling cooperation, not only by implementing punishment against free-riders [6–8], but also by means of their visibility, which enables agents to condition their actions on whether these institutions are present or not.

Punishment provides a possible solution to the problem of collective action [9–14]. The vast majority of theoretical and experimental work focuses on pro-social peer punishment, exerted by peers and individually directed towards those who do not cooperate [15–20]. Anti-social peer punishment is instead directed towards those who do contribute to the public good [21]. Experiments and models have shown that anti-social punishment can diminish the effectiveness of punishment in promoting cooperation [22–25]. In many instances, however, punishment is not individual, but a coordinated action of many individuals [26]. An extreme form of such coordination is the kind of pool punishment that emerged as the typical way of punishment in modern human societies: individuals commit an investment into a pool to pay for the punishment of those who do not comply with a social norm [6,27–29]. Here, we show that pro-social punishment can withstand the presence of cheaters and anti-social behaviour, but this outcome only emerges when considering *all* possible strategies in a public goods game. This result highlights the importance of avoiding artificial restrictions in the strategy set of evolutionary models.

We study the evolutionary dynamics of coordinated anti-social punishment and ask whether the associated coordinated punishment can emerge, potentially undermining cooperation. Our model allows for the evolutionary competition between anti-social and pro-social punishment. We consider two main scenarios. (i) Observable institutions allow individuals to condition their actions on the existence of punishment institutions. (ii) If institutions cannot be observed, individuals are unable to condition their behaviour. When punishment is not observable, anti-social punishment triggers a collapse of the public good. If institutions are observable, cooperation can be established and stabilized by pro-social punishment, even in the face of anti-social behaviour.

## 2. The model

### 2.1. A game of cooperation with institutional punishment

Our model follows Sigmund *et al.* [6] in the basic set-up of an optional public goods game between *n* players with three stages. (i) The first stage is institutional commitment, in which players may commit funds to an institution that will later punish free-riders or cooperators. (ii) The second stage is the public goods game, in which individuals may decide whether to contribute or not to a public good. (iii) The third stage is punishment, in which players are fined in accordance to the institutions in place.

In the institutional commitment stage (i), participants choose what kind of institution they want to support. They can support pro-social punishment directed to defectors, or anti-social punishment of cooperators, both, or none. Funding an institution costs a fixed amount *γ* if the punishment takes place. An institution is established—and therefore costly—only if there are at least *k* players contributing to it.

(ii) During the public goods stage, players use the information about the institutions in place, choosing whether they contribute an amount *c* > 0. Contributions are multiplied by a factor *r* > 1 and distributed among the *n* − 1 other players [30].

(iii) During the punishment stage, agents are fined according to the institutions in place, and the amount of players supporting the corresponding institutions. Non-contributors are punished by an amount *β* multiplied by the number of supporters of the pro-social institution. Contributors are punished by an amount *β* multiplied by the number of supporters of the anti-social pool.

Since the game is optional, we also let agents opt out of the game altogether. Those that do not take part in the game obtain a loner pay-off *σ* > 0, regardless of the decisions of others [31]. An optional game, therefore, includes the ‘loner’ strategy, whereas a non-optional game precludes it.

**Figure 1.**
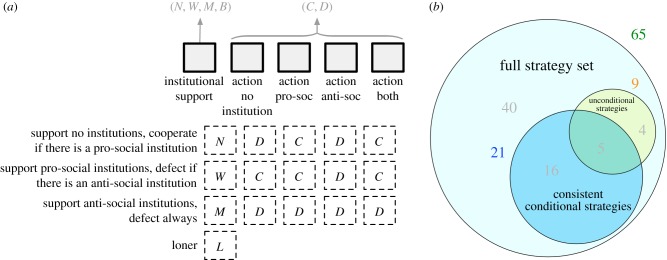
Description of the strategy sets. (*a*) The 64 strategies taking part in the game are characterized by their contribution to the punishment pool (none (*N*), pro-social (*W*), anti-social (*M*) or both (*B*)) and their action for each institutional set-up. In addition, in an optional game, we have the loner strategy which neither punishes nor gets punished. (*b*) The 65 strategies can be reduced to 21 by assuming consistent actions—players do not support pools that punish them. Alternatively, one can focus on nine unconditional strategies, where actions are not affected by the punishment set-up. Iterated removal of dominated strategies results in five strategies that are also consistent. (Online version in colour.)

When agents can decide what to do depending on the existence of punishment institutions, a large strategy set emerges, as follows. In the first stage of the game, an individual decides their institutional support; with options for supporting no institution (*N*), only a pro-social institution (*W*), only an anti-social institution (*M*) or both institutions (*B*)—thus institutional support entails four possibilities. In addition, agents also decide whether to contribute or not to the public good, contingent on the institutional arrangement in place; i.e. cooperate or defect given there is no institution, cooperate or defect when there is a pro-social institution only, what to do when there is only an anti-social institution, and what to do when both institutions are in place—thus, we have 2^4^ possibilities. This yields in total 4 × 2^4^ = 64 strategies. If we further make the game optional and include the loner option, we obtain a total of 65 strategies (figure 1*a*).

In the analysis that follows we will focus on different subsets of this large strategy set. These subsets will also imply different assumptions in the game. In particular, we study a set of nine non-conditional strategies, in which cooperation or defection does not depend on institutional arrangements—this is equivalent to institutions that cannot be observed. We also study the set of all 65 strategies, whose analysis will be shown equivalent to that arising from the set of 21 consistent strategies. A consistent strategy is such that a player will not contribute to an institution that would punish her actions (figure 1*b*).

### 2.2. Evolutionary dynamics

The game described above determines the pay-off of each player in the population. We calculate the average pay-off across all possible configurations of groups given the current numbers of each type in the population, such that all players using the same strategy have the same pay-off *π*. This pay-off determines how many players will adopt the corresponding strategy, as successful strategies spread in a finite population in proportion to their relative fitness. We consider a Moran process, where a single individual chooses a new strategy in each time step with probability proportional to fitness *f*. We assume an exponential pay-off to fitness mapping [32,33], such that fitness is given by *f* = exp [+*ωπ*], where *ω* is the intensity of selection; see electronic supplementary material for details. In addition, there is a small probability *μ* that an individual switches to a new random type. In our simulations, we focus on the case of population size *N* = 50, mutation rate *μ* = 0.001, and intensity of selection *ω* = 10.

## 3. Results

We study which strategies are favoured by an evolutionary process. Under small mutation rates, the dynamics of the evolutionary process is confined to edges between two strategies [34,35]. Therefore, it is instructive to first compare pay-offs between any two strategies. A full overview of the (1/2) × 65 × 64 = 2080 strategy pairs is possible, but hard to grasp. We thus start by reducing the size of this large strategy set, making further assumptions on the nature of possible strategies.

### 3.1. Non-observable institutions

**Figure 2.**
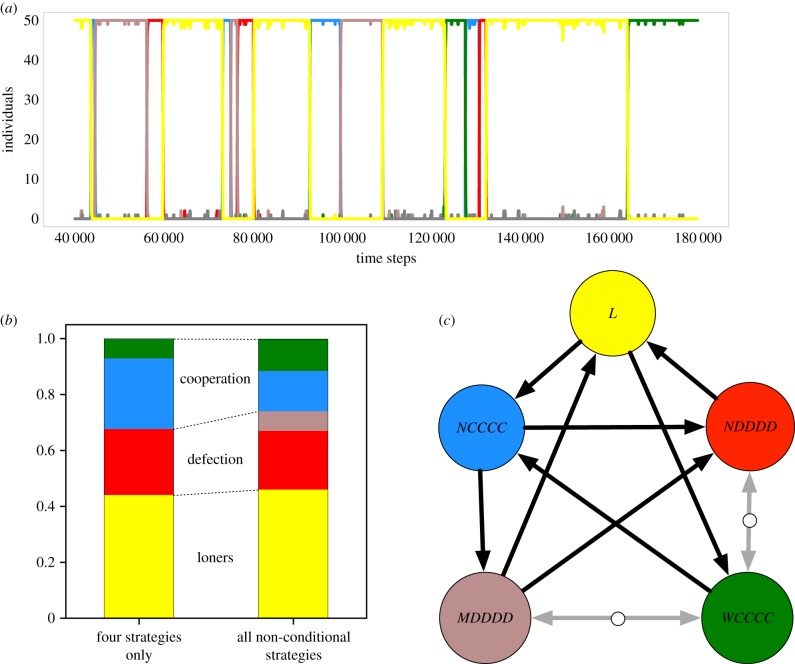
Evolutionary dynamics for the nine unconditional strategies. (*a*) Typical simulation run over 140 000 time steps shows different cycles in which strategies replace each other, the dominated strategies that punish themselves are only present in low abundance (colour code for the strategies as in the other panels). Because of the low mutation rate, the abundances are typically close to 0% or 100%. (*b*) The stationary distribution obtained from computer simulations—four strategies from [6] only, without anti-social punishment, versus all unconditional strategies. Averages are taken over 300 independent repetitions, each running for 5 × 10^6^ generations, averaging the second half of each replicate. The inclusion of the anti-social institution (*MDDDD*), which from the outside is paradoxical and should play no role in evolution, reduces the level of cooperation. (*c*) Pairwise invasion diagram for the five strategies that are not dominated. Circles represent the strategies, arrows indicate the direction of selection. Bold arrows represent the paths that are prevalent in computer simulations. The dynamics can follow several intertwined cycles, e.g. *L* → *NCCCC* → *MDDDD* → *NDDDD* → *L*; see main text (we use our default set of game parameters *n* = 5, *σ* = 1, *c* = 1, *r* = 3, *γ* = 0.7, *β* = 1.5, population size *N* = 50, mutation rate *μ* = 0.001, intensity of selection *ω* = 10). (Online version in colour.)

In the simplest case, individuals cannot condition their actions on the existence of a punishment institution [6]. This is equivalent to punishment institutions that cannot be observed. This case leads to nine strategies, as follows. Individuals have four options to support institutions multiplied by two possible actions in the public goods game. In addition, individuals can choose to abstain from the game. Out of these nine strategies, four are dominated by others. However, instead of neglecting these strategies from the beginning, we include them in our computational model and let evolution decide whether they play any role. Pro-social institutions can promote temporary cooperation, even when fines are exclusively directed towards defectors and not used to stabilize punishment [6]. But this kind of model assumes that anti-social institutions are excluded. When allowing for anti-social institutions, cooperation can not only be undermined by defectors not supporting any institution but also by defectors that in addition set up an anti-social punishment institution and stretch their relative advantage (figure 2). As a consequence, more players tend to abstain from the public goods game and—more importantly—fewer players cooperate. Thus, anti-social institution supporters temporarily invade. This dynamics triggers a sizeable reduction in cooperation as shown in figure 2.

Figure 2 also shows two unstable fixed points: between the pro-social and the anti-social institution (*WCCCC* and *MDDDD*), and between the pro-social institution and defectors (*WCCCC* and *NDDDD*). The existence of these unstable fixed points can be illustrated from the competition between the two associated strategies. In the case of *WCCCC* and *NDDDD*, the associated pay-offs in a population with *j* cooperating players are

3.1 $$\begin{array}{rl} \pi_{WCCCC} & =-\gamma -c+\sum_{i=0}^{n-1}\frac{\left(\begin{array}{c} \;j-1\\ i \end{array}\right)\left(\begin{array}{c} N-j\\ n-i-1 \end{array}\right)}{\left(\begin{array}{c} N-1\\ n-1 \end{array}\right)}\frac{cri}{n-1}\\ & =-\gamma -c+cr\frac{\;j-1}{N-1} \end{array}$$

and

3.2 $$\begin{array}{rl} \pi_{NDDDD} & =+\sum_{i=0}^{n-1}\frac{\left(\begin{array}{c} \;j\;\\ i \end{array}\right)\left(\begin{array}{c} N-j-1\\ n-i-1 \end{array}\right)}{\left(\begin{array}{c} N-1n-1 \end{array}\right)}\left(\frac{cri}{n-1}-\beta i\right)\\ & =(cr-\beta (n-1))\frac{\;j}{N-1}. \end{array}$$

For *j* = 1, we have *π*_*WCCCC*_ < *π*_*NDDDD*_ when the costs of cooperation and supporting the institution outweigh the fine imposed on the defectors and the additional benefit they get from the public goods (due to the setting where a cooperating player does not benefit from her own contribution), −*γ* − *c* < (*cr* − *β*(*n* − 1)/*N* − 1). This condition will always be fulfilled for large *N*. In this case, defectors cannot be invaded by cooperating supporters of a pro-social institution. For *j* = *n* − 1, the condition for *π*_*WCCCC*_ > *π*_*NDDDD*_ reduces to +*γ* + *c* < *β*(*n* − 1), i.e. the costs of cooperation and supporting the institution must be smaller than the fine imposed on the defectors. Thus, cooperating supporters of a pro-social institution cannot be invaded by defectors. Since neither of the two strategies can invade the others (and since the pay-offs are linear in *j*), this results in a bi-stability.

A similar argument holds for the pair *MDDDD* and *WCCCC*. A more comprehensive (numerical) analysis that includes all pairs of these nine strategies is presented in the electronic supplementary material.

The paths via such bi-stabilities are not prevalent in the computer simulations that will typically follow the paths highlighted in the figure. Four different cycles are prevalent: the first one, *L* → *NCCCC* → *NDDDD* → *L*, has already been described in Hauert *et al.* [31]; the second one, *L* → *WCCCC* → *NCCCC* → *NDDDD* → *L*, additionally emerges in the institutional punishment model of Sigmund *et al.* [6]; while the remaining two, *L* → *NCCCC* → *MDDDD* → *L* and *L* → *NCCCC* → *MDDDD* → *NDDDD* → *L*, emerge only in the presence of anti-social punishment institutions.

One may argue that an anti-social institution should never arise because cooperators can be invaded by defectors through an easier path without any anti-social punishment. However, this argument also applies to the transition from loners to cooperators, which could occur with a pro-social pool, but also without any punishment. The crucial difference is that, while the pro-social pools can rise to high abundance [6], the anti-social behaviour only plays an important role in facilitating the emergence of other strategies without becoming prevalent itself. The complexity of implementing an anti-social and a pro-social institution per se is the same. Their asymmetry arises only from evolutionary competition.

Notably, figure 2*b* shows that artificially taking out seemingly unimportant strategies has an effect on the predicted level of cooperation. For our default set of parameters, we observe a slight increase in loners, from 44% to 46%. Likewise, defection increases by about 4% and the overall level of cooperation decreases by 6% through the introduction of the anti-social punishment institution.

### 3.2. Observable institutions

In many cases, information on punishment pools may be available before players need to make a decision on their contribution, such that players can condition their actions on the existence of institutions [26,28,29]. For example, criminals are arguably less likely to offend if they know an institution is in place to punish them [36]—although see also [37]. First, we focus on consistent conditional strategies (figure 1) which do not punish themselves: if individuals support a pro-social institution, they cooperate if that institution is in place. If individuals support an anti-social institution, they defect if that institution is in place. Thus, they do not support both institutions at the same time. Moreover, if they cooperate (defect) under a single institution, they also cooperate (defect) when both institutions exist. This set contains four strategies that support the pro-social institution, *W* ○ *C* ○ *C* (where the entries ○ are either *C* or *D*), and four strategies that support the anti-social institution, *M* ○ ○ *DD*. In addition, we have 12 strategies that do not support any institution, *N* ○ ○ ○ ○, where the four strategies *N* ○ *CCD* and *N* ○ *DDC* are excluded. Finally, we have the option to abstain from the public goods game, *L*.

Electronic supplementary material, figure S1 summarizes the dynamics between the associated 210 (=(1/2) × 21 × 20) pairs of consistent conditional strategies. In this set of strategies, no strategy is strictly dominated. The evolutionary dynamics is governed by stable coexistences between a minority of players that support the pro-social institution, *W* ○ *C* ○ *C* (called *I* below), and opportunists that cooperate only when the pro-social institution is in place [28], *NDC* ○ ○ (called *O* below). As long as a single supporter of the institution can induce its existence, *k* = 1, their pay-off is *π*_*I*_ = *r c* − *c* − *γ*. The probability that a focal opportunist is in a group that contains at least one supporter of the pro-social institution is 1 − *x*^*n*−1^, where *x* is the fraction of opportunists, who obtain a pay-off *rc* − *c* in that case. If opportunists are alone, no one cooperates and their pay-off is zero, such that their average pay-off becomes *π*_*O*_ = (*rc* − *c*)(1 − *x*^*n*−1^). The condition *π*_*I*_ = *π*_*O*_ leads to a unique stable equilibrium,

3.3 $$x^{*}={\left(\frac{\gamma }{rc-c}\right)}^{1/(n-1)}.$$

**Figure 3.**
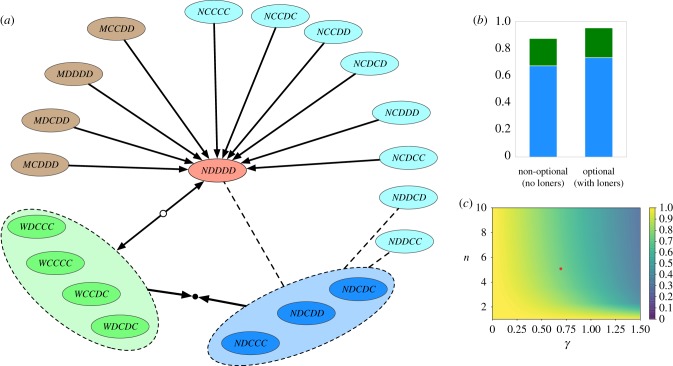
Evolutionary dynamics for the 20 consistent conditional strategies. (*a*) Evolutionary dynamics between pairs of strategies. We only depict invasions that are prevalent in the dynamics under strong selection. Given sufficient time, stable coexistences between two kinds of players emerge: those that support pro-social punishment and those not supporting any institution, but cooperating in the presence of pro-social punishment. For simplicity, the diagram focuses on a non-optional game: adding the loner strategy creates a fast path from defection, via loners, into either side of the stable mixture. (*b*) There are 12 different stable coexistences with similar abundance based on the four strategies supporting pro-social punishment and the three opportunistic strategies that cooperate in the presence, but defect in the absence, of coordinated pro-social punishment. We group these strategies in the cases of non-optional and optional public goods games. Averages taken as described in figure 2. (*c*) The probability that a pro-social pool is implemented in the coexistence decreases with increasing costs *γ* and increasing group size *n*. The dot indicates our default set of parameters (see main text or figure 2). (Online version in colour.)

The probability that an institution is implemented in a group is then 1 − (x*)^*n*^, i.e. a small fraction of supporters of the institution can induce high levels of cooperation (figure 3).

The resulting stable coexistences are remarkably resilient to evolutionary invasions. We can see this by doing a pairwise analysis, and considering strategies *W* ○ *C* ○ *C* and *NDC* ○ ○, where the entry in ○ is irrelevant (and the last entry in *W* ○ *C* ○ *C* follows from our restriction to consistent strategies). This coexistence cannot be invaded by any other single mutant.

— Players that cooperate in the absence of an institution, *NCC* ○ ○, would be exploited by the *NDC* ○ ○ resident.
— Those that defect in the presence of an institution, *NDD* ○ ○, would suffer from punishment.
— Any player supporting the anti-social institution would obtain a lower pay-off than the players in the stable coexistence: there, both players obtain −*γ* + *rc* − *c*. A single supporter of the anti-social institution would at most get −*γ* + *r c* − *β*. This assumes that, in the presence of both institutions, all the other players cooperate and that at least one individual contributed to a pro-social institution. As *β* > *c*, this pay-off is always smaller than the pay-off of the two resident types. Therefore, supporters of an anti-social institution cannot invade.

Thus, the coexistence between *W* ○ *C* ○ *C* and *NDC* ○ ○ is stable against single mutants. As we have made no assumption on ○, this holds for all such strategies. There is no other pairwise stable coexistence in the system.

Although this theoretical analysis assumes that populations are very large, our simulation results for *N* = 50 perfectly match the prediction (figure 3*b*). This is due to the fact that selection is strong in the simulations. As a general rule of thumb, we expect this prediction to hold whenever the product of intensity of selection and population size is large [38]. This relationship between infinite and finite populations has been studied in detail elsewhere; see [39].

Note that, with conditional strategies, defection can be left via neutral paths towards strategies that do not support any institution and do not cooperate in the absence of institutions—but potentially in their presence. This implies that the game no longer needs to be optional for cooperation to evolve [28], a potential issue with previous models [40]. Figure 3 shows the strategies in this set of 20 strategies as well the typical evolutionary dynamics between pairs of strategies.

Our model assumes that, if it is costly to make institutions visible, the cost is part of the funds paid in order to establish the institution. However, it is also possible to assume that this cost is paid by strategies using conditional information. This extension is discussed in the electronic supplementary material.

### 3.3. Including all conditional strategies

**Figure 4.**
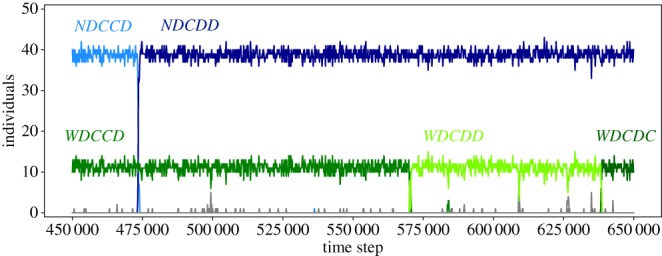
Time series showing a snapshot of the evolution in the complete strategy space of 65 strategies. Different instances of stable coexistences in the form *WDC* ○ *C* and *NDC* ○ ○ are following each other, where each strategy can be replaced independently of the other. For instance, here *NDCCD* is replaced by *NDCDD* before replacements within the institutional supporters take place (from *WDCCD* via *WDCDD* to *WDCDC*). For the present parameter set (see main text), 23% of institutional supporters induce cooperation in 73% of all games (window over 2 × 10^5^ time steps, *k* = 1). (Online version in colour.)

So far, we have given arguments that allow us to focus on specific subsets of strategies. To verify the robustness of our findings, we also consider the full strategy set of 65 strategies and implement the same computational evolutionary model as before. Because this strategy space includes many new possibilities, a plethora of different combinations could evolve. However, the same coexistence between individuals supporting pro-social institutions and opportunists not supporting any institution is found again, with the same 12 stable coexistences as in the subset of the 20 consistent strategies considered above. Thus, the evolutionary outcome is independent of the choice of the strategy subset in our model once the key opportunistic strategies are considered. Figure 4 shows a typical time series of the dynamics with a complete strategy space. The system spends the vast majority of time in stable coexistences, where the strategies are occasionally replaced by others that display the same behaviour in this situation.

### 3.4. Higher thresholds for punishment implementation

When a certain number of supporters is required to implement an institution, all players can find themselves in groups with no punishment in place. This implies that the behaviour in such situations is under selection. Qualitatively, the results remain identical to the case where punishment can be implemented by a single supporter, *k* = 1, but the position of the fixed point and the level of cooperation can change. The structure of the game resembles a threshold public goods game [41,42]; see electronic supplementary material. When at least two supporters of an institution are needed, *k* = 2, the equilibrium fraction of players supporting the pro-social set-up increases from ≈23% (*k* = 1) to ≈43% (*k* = 2). At the same time, the probability that the pool is actually implemented decreases slightly from ≈73% (*k* = 1) to ≈72%. A pairwise analysis of strategies reveals that now other coexistences are possible as well, for example between *WCCDD* and *NDCDD* (see figure 1 for an explanation of the strategy notation). In the electronic supplementary material, we show that, for *k* = 2, all these additional coexistences are unstable with respect to the invasion of a third strategy. Only the coexistences discussed above for the case of *k* = 1 remain stable against all invasions.

The equilibrium that sustains cooperation resembles that arising in a volunteer’s dilemma [43], where the volunteering threshold, *k*, represents the number of contributors required to establish a punishment institution. We also note that the possibility of these types of coexistences has been discussed in the context of nonlinear public good games [44]. Here, we also show that this type of coexistence is particularly resilient and can arise with many flavours in the context of pool punishment, i.e. different combinations of conditional strategies that can resist invasions arising from a large strategy space. Our simulations also show that this equilibrium is stable when the population is finite and includes demographic noise.

## 4. Discussion

We find that pro-social, but not anti-social, punishment emerges based on individual-level selection with full symmetry between the two kinds of punishment institutions. Evolutionary dynamics introduces a symmetry breaking between the two kinds of strategies selected that favours pro-social states. The prevalent outcome is a stable coexistence between cooperators supporting a pro-social pool and those willing to cooperate when such institutions are in place, but not cooperating otherwise. For peer punishment, such stable coexistences between strategies do not appear, because they require an honest signal prior to the game [45,46].

Typical models in evolutionary game theory restrict themselves to a small number of strategies that seem to be interesting from the outset, which greatly facilitates the analysis [47]. While such a restriction can be highly insightful, the conclusions from the model can in some cases strongly depend on the strategy set [23,24,48–50]. Our computational model implements the entire possible set of strategies in the context of coordinated, institutional punishment. One may be tempted to exclude strategies that seem illogical in the context of the model, but this can be misguided. Selecting a behaviour necessarily implies that other behaviours are driven out. Therefore, the absence of behaviour should be the result of evolutionary competition and not a result of the modeller’s subjective choice. As shown in this paper, strategies can have a strong impact on the evolutionary outcome even if they do not rise to high average abundances and only temporarily pave the way for other strategies. We believe this robustness test is important in simple models, which may otherwise have biased conclusions.

In our model, the maintenance of pro-social punishment relies on a small minority which supports them, but their robustness stems from the fact that they are constantly challenged by the presence of players that stop cooperating in the absence of pro-social institutions. This combination is empirically prevalent, with resilient public goods often being supported by a minority of contributors [51]. The key to the maintenance of the public good is the observability of institutions: only public knowledge of the presence of punishment institutions allows the conditional strategies that ultimately prevent the rise of anti-social behaviour, either in the form of defection or in the form of coordinated anti-social punishment institutions that never rise to high abundance, but can undermine the public good.

An interesting problem arising here is the possible effect of institutional asymmetries. For example, anti-social institutions may entail higher costs than pro-social ones, or implement asymmetric fines in which contributors and non-contributors are punished differently. In particular, anti-social institutions may offer an evolutionary advantage if they level intrinsic asymmetries between players.

Our model is primarily concerned with the emergence and establishment of primitive institutions, thus we do not explicitly model implementation details. Instead, we assume that, once implemented, institutions for punishment will work as expected. In reality, additional issues may also arise, e.g. due to corruption [8,52,53] or group heterogeneity [54]. Asymmetries, in particular, have been shown to be important in infinite populations [55]. The tools necessary to study finite evolutionary dynamics arising from asymmetric games are not fully developed yet [56].

Most modern societies put law enforcement into the hands of institutions and do not allow their citizens to punish others directly. Our model suggests that our instinct against taking the law into our own hands is justified: the value of the signal conferred by the presence of pro-social punishment institutions may be crucial in promoting the kind of cooperation observed in humans.

## Supplementary Material

Supplementary text

## Data accessibility

Our simulation code is available at https://osf.io/6tjmb/.
