# Supplementary material for: Evolution of coordinated punishment to enforce cooperation from an unbiased strategy space
Source: J R Soc Interface. 2019 Jul 24;16(156):20190127. doi: 10.1098/rsif.2019.0127 (PMC6685031; doi:10.1098/rsif.2019.0127)
Supplement: Supplementary text [file rsif20190127supp1.pdf]

Electronic Supplementary Material: Evolution of coordinated punishment to enforce cooperation from an unbiased strategy space

Julián García and Arne Traulsen

A Evolutionary Dynamics

We assume that successful strategies spread in a finite population in proportion to their relative fitness. For each one of the strategy sets, we consider a Moran process with exponential payoff to fitness mapping [1, 2]:

- We calculate the average payoff π of each individual given the current composition of the population. Fitness is then defined as $f = \exp[+\omega\pi]$
- One individual is selected at random, proportional to fitness. With probability $1 - \mu$, the offspring is identical to the parent. With probability μ, its offspring is mutated and is assigned a new random type (selected among all types, including the parents).
- The offspring replaces a randomly chosen individual.

An analytical approach based on small mutation rates [3] is problematic for the present model, as there are stable mixtures of strategies which call for mutation rates exponentially small in the population size and the intensity of selection [4]. Also a weak selection analysis for any mutation rate makes limited sense in our case [5], as this is based on deviations from a state where a large number strategies would coexist at identical abundance – a situation that appears ill-fitted to our setup with a large number of strategies. To bypass these difficulties, we focus on simulations to single out the important parts of the dynamics, which we then study analytically.

B Parameter choice

For the evolutionary dynamics, we assume a population of size $N = 50$, a mutation rate $\mu = 10^{-3}$, and a high intensity of selection $\omega = 10$ to obtain results robust under changes of the parameters of the game [6, 7]. Note that the model is interesting only if these parameters fulfill the following four conditions

(i) A group in which all individuals contribute to a pro-social pool cooperates and is better off than a group in which everyone abstains, i.e., $rc - c - \gamma > \sigma$.

(ii) It is better to abstain than to be in a group of defectors, $\sigma > 0$.

(iii) The fine exceeds the costs, $\beta > c$, i.e., punishment is always a deterrent, since the fine paid for not contributing outweighs contributions themselves.

(iv) The fine exceeds the cost of punishing someone, $\beta > \gamma$ and the cost of contribution is smaller than the cost of punishing, $\gamma < c$.

For our simulations, the game parameters are as chosen as follows: The loner payoff is $\sigma = 1$. Contribution to the public good is $c = 1$, and the multiplier is $r = 3$. The individual cost of supporting a sanctioning pool is $\gamma = 0.7$. A fine per unit of contribution to a sanctioning pool is $\beta = 1.5$. In our simulations, we work with a population size of $N = 50$ and a group size of $n = 5$.

C Analysis of pairwise coexistences for $k = 1$

C.1 Pairwise coexistences for k=1.

NCCDC	– – – –	NCCCC	NDCCC	⟵	NCCDD	WDCDC	⟵ ○ ⟶	NCDCD	WDCDC	⟶ ● ⟵	NDCDC	WDCDC	⟵ ○ ⟶	NDDDD
NCCDD	– – – –	NCCCC	NDCDC	⟵	NCCDD	MCCDD	⟵	NCDCD	MCCDD	⟶	NDCDC	MCCDD	⟶	NDDDD
NCDCC	– – – –	NCCCC	NDCDD	⟵	NCCDD	MCDDD	⟵	NCDCD	MCDDD	⟶	NDCDC	MCDDD	⟶	NDDDD
NCDCD	– – – –	NCCCC	NDDCC	⟵	NCCDD	MDCDD	⟵	NCDCD	MDCDD	⟶	NDCDC	MDCDD	⟶	NDDDD
NCDDD	– – – –	NCCCC	NDDCD	⟵	NCCDD	MDDDD	⟵	NCDCD	MDDDD	⟶	NDCDC	MDDDD	⟶	NDDDD
NDCCC	⟵	NCCCC	NDDDD	⟵	NCCDD	L	⟶	NCDCD	L	⟵	NDCDC	L	⟵	NDDDD
NDCDC	⟵	NCCCC	WCCCC	⟶	NCCDD	NDCCC	⟵	NCDDD	NDDCC	– – – –	NDCDD	WCCDC	– – – –	WCCCC
NDCDD	⟵	NCCCC	WCCDC	⟶	NCCDD	NDCDC	⟵	NCDDD	NDDCD	– – – –	NDCDD	WDCCC	– – – –	WCCCC
NDDCC	⟵	NCCCC	WDCCC	⟶	NCCDD	NDCDD	⟵	NCDDD	NDDDD	– – – –	NDCDD	WDCDC	– – – –	WCCCC
NDDCD	⟵	NCCCC	WDCDC	⟶	NCCDD	NDDCC	⟵	NCDDD	WCCCC	⟶ ● ⟵	NDCDD	MCCDD	⟵ ○ ⟶	WCCCC
NDDDD	⟵	NCCCC	MCCDD	⟶	NCCDD	NDDCD	⟵	NCDDD	WCCDC	⟶ ● ⟵	NDCDD	MCDDD	⟵ ○ ⟶	WCCCC
WCCCC	⟶	NCCCC	MCDDD	⟶	NCCDD	NDDDD	⟵	NCDDD	WDCCC	⟶ ● ⟵	NDCDD	MDCDD	⟵ ○ ⟶	WCCCC
WCCDC	⟶	NCCCC	MDCDD	⟶	NCCDD	WCCCC	⟵ ○ ⟶	NCDDD	WDCDC	⟶ ● ⟵	NDCDD	MDDDD	⟵ ○ ⟶	WCCCC
WDCCC	⟶	NCCCC	MDDDD	⟶	NCCDD	WCCDC	⟵ ○ ⟶	NCDDD	MCCDD	⟶	NDCDD	L	⟶	WCCCC
WDCDC	⟶	NCCCC	L	⟶	NCCDD	WDCCC	⟵ ○ ⟶	NCDDD	MCDDD	⟶	NDCDD	WDCCC	– – – –	WCCDC
MCCDD	⟵	NCCCC	NCDCD	– – – –	NCDCC	WDCDC	⟵ ○ ⟶	NCDDD	MDCDD	⟶	NDCDD	WDCDC	– – – –	WCCDC
MCDDD	⟵	NCCCC	NCDDD	– – – –	NCDCC	MCCDD	⟶	NCDDD	MDDDD	⟶	NDCDD	MCCDD	⟵ ○ ⟶	WCCDC
MDCDD	⟵	NCCCC	NDCCC	⟵	NCDCC	MCDDD	⟶	NCDDD	L	⟵	NDCDD	MCDDD	⟵ ○ ⟶	WCCDC
MDDDD	⟵	NCCCC	NDCDC	⟵	NCDCC	MDCDD	⟶	NCDDD	NDDCD	– – – –	NDDCC	MDCDD	⟵ ○ ⟶	WCCDC
L	⟶	NCCCC	NDCDD	⟵	NCDCC	MDDDD	⟶	NCDDD	NDDDD	– – – –	NDDCC	MDDDD	⟵ ○ ⟶	WCCDC
NCCDD	– – – –	NCCDC	NDDCC	⟵	NCDCC	L	⟶	NCDDD	WCCCC	⟵ ○ ⟶	NDDCC	L	⟶	WCCDC
NCDCC	– – – –	NCCDC	NDDCD	⟵	NCDCC	NDCDC	– – – –	NDCCC	WCCDC	⟵ ○ ⟶	NDDCC	WDCDC	– – – –	WDCCC
NCDCD	– – – –	NCCDC	NDDDD	⟵	NCDCC	NDCDD	– – – –	NDCCC	WDCCC	⟵ ○ ⟶	NDDCC	MCCDD	⟵ ○ ⟶	WDCCC
NCDDD	– – – –	NCCDC	WCCCC	⟵ ○ ⟶	NCDCC	NDDCC	– – – –	NDCCC	WDCDC	⟵ ○ ⟶	NDDCC	MCDDD	⟵ ○ ⟶	WDCCC
NDCCC	⟵	NCCDC	WCCDC	⟵ ○ ⟶	NCDCC	NDDCD	– – – –	NDCCC	MCCDD	⟵	NDDCC	MDCDD	⟵ ○ ⟶	WDCCC
NDCDC	⟵	NCCDC	WDCCC	⟵ ○ ⟶	NCDCC	NDDDD	– – – –	NDCCC	MCDDD	⟵	NDDCC	MDDDD	⟵ ○ ⟶	WDCCC
NDCDD	⟵	NCCDC	WDCDC	⟵ ○ ⟶	NCDCC	WCCCC	⟶ ● ⟵	NDCCC	MDCDD	⟵	NDDCC	L	⟶	WDCCC
NDDCC	⟵	NCCDC	MCCDD	⟵	NCDCC	WCCDC	⟶ ● ⟵	NDCCC	MDDDD	⟵	NDDCC	MCCDD	⟵ ○ ⟶	WDCDC
NDDCD	⟵	NCCDC	MCDDD	⟵	NCDCC	WDCCC	⟶ ● ⟵	NDCCC	L	⟵	NDDCC	MCDDD	⟵ ○ ⟶	WDCDC
NDDDD	⟵	NCCDC	MDCDD	⟵	NCDCC	WDCDC	⟶ ● ⟵	NDCCC	NDDDD	– – – –	NDDCD	MDCDD	⟵ ○ ⟶	WDCDC
WCCCC	⟶	NCCDC	MDDDD	⟵	NCDCC	MCCDD	⟵	NDCCC	WCCCC	⟵ ○ ⟶	NDDCD	MDDDD	⟵ ○ ⟶	WDCDC
WCCDC	⟶	NCCDC	L	⟶	NCDCC	MCDDD	⟵	NDCCC	WCCDC	⟵ ○ ⟶	NDDCD	L	⟶	WDCDC
WDCCC	⟶	NCCDC	NCDDD	– – – –	NCDCD	MDCDD	⟵	NDCCC	WDCCC	⟵ ○ ⟶	NDDCD	MCDDD	– – – –	MCCDD
WDCDC	⟶	NCCDC	NDCCC	⟵	NCDCD	MDDDD	⟵	NDCCC	WDCDC	⟵ ○ ⟶	NDDCD	MDCDD	– – – –	MCCDD
MCCDD	⟶	NCCDC	NDCDC	⟵	NCDCD	L	⟵	NDCCC	MCCDD	⟵	NDDCD	MDDDD	– – – –	MCCDD
MCDDD	⟶	NCCDC	NDCDD	⟵	NCDCD	NDCDD	– – – –	NDCDC	MCDDD	⟵	NDDCD	L	⟵	MCCDD
MDCDD	⟶	NCCDC	NDDCC	⟵	NCDCD	NDDCC	– – – –	NDCDC	MDCDD	⟵	NDDCD	MDCDD	– – – –	MCDDD
MDDDD	⟶	NCCDC	NDDCD	⟵	NCDCD	NDDCD	– – – –	NDCDC	MDDDD	⟵	NDDCD	MDDDD	– – – –	MCDDD
L	⟶	NCCDC	NDDDD	⟵	NCDCD	NDDDD	– – – –	NDCDC	L	⟵	NDDCD	L	⟵	MCDDD
NCDCC	– – – –	NCCDD	WCCCC	⟵ ○ ⟶	NCDCD	WCCCC	⟶ ● ⟵	NDCDC	WCCCC	⟵ ○ ⟶	NDDDD	MDDDD	– – – –	MDCDD
NCDCD	– – – –	NCCDD	WCCDC	⟵ ○ ⟶	NCDCD	WCCDC	⟶ ● ⟵	NDCDC	WCCDC	⟵ ○ ⟶	NDDDD	L	⟵	MDCDD
NCDDD	– – – –	NCCDD	WDCCC	⟵ ○ ⟶	NCDCD	WDCCC	⟶ ● ⟵	NDCDC	WDCCC	⟵ ○ ⟶	NDDDD	L	⟵	MDDDD

ESM Figure 1: In an infinite population, a classification between all pairs of consistent conditional strategies as neutral (– – – –), dominance (⟶ or ⟵), bistabilities (⟵ ○ ⟶), or coexistences (⟶ ● ⟵) is possible. These results do not change with the parameters, but require $k = 1$. If the population size is no longer infinite, but large compared to the group size, the classification also determines the evolutionary dynamics in finite populations via fixation probabilities and fixation times.

As discussed in the main text, when a single individual can trigger the existence of a pool, $k = 1$, all pairwise stable coexistences between opportunists and supporters of the

pro-social institution display the same behaviour. Supporters of the pro-social pool always find themselves in fully cooperative groups, leading to a payoff

$$\pi_I = rc - c - \gamma. \tag{1}$$

To simplify the analysis, we assume that the population is so large that we can assume it is infinite. Then, the probability that an opportunist faces a group with at least one supporter of the pro-social pool is $1 - x^{n-1}$, where x is the fraction of opportunists, who obtain a payoff $rc - c$ in that case. If opportunists are alone, no one cooperates and their payoff is zero. On average, this leads to a payoff

$$\pi_O = (rc - c)\left(1 - x^{n-1}\right) \tag{2}$$

In the unique stable equilibrium x^* of the associated replicator dynamics [8], both payoffs must be equal, leading to

$$x^* = \left(\frac{\gamma}{rc - c}\right)^{1/(n-1)}. \tag{3}$$

Supporters of pro-social sanctions can invade opportunists (as they immediately induce cooperation) and vice versa (as opportunists save the cost γ). For $k = 1$, the game is identical to a volunteer's dilemma [9, 10, 11].

D Pairwise coexistences for $k > 1$

Let us focus on the coexistences between supporters of the sanctioning pools and opportunists, $NDC \circ \circ$, in the case where more than one individual is needed to implement punishment, $k > 1$. We assume that only effective pools that are actually realised cause any costs.

We assume that supporters of the pool defect if the pool is not implemented successfully, i.e. we focus on strategies $WDC \circ C$ (the strategies $WCC \circ C$ can be invaded by $WDC \circ C$ for $k > 1$). We abbreviate the payoff of the $WDC \circ C$ strategies as

$$\pi_{ID} = (rc - c - \gamma) \sum_{j=k-1}^{n-1} \binom{n-1}{j} (1-x)^j x^{n-1-j}, \tag{4}$$

where the sum captures the probability that a pool is realised — otherwise their payoff is zero. The opportunists obtain

$$\pi_O = (rc - c) \sum_{j=k}^{n-1} \binom{n-1}{j} (1-x)^j x^{n-1-j}. \tag{5}$$

Equating the payoffs, $\pi_{ID} = \pi_O$, leads to an expression for the fixed point,

$$\frac{\gamma}{rc - c} = \frac{\binom{n-1}{k-1} x^{n-k} (1-x)^{k-1}}{\sum_{j=k-1}^{n-1} \binom{n-1}{j} x^{n-j-1} (1-x)^j} \tag{6}$$

The right hand side of this equation increases monotonously from 0, for $x = 0$, to 1 for $x = 1$, see Peña *et al.* [12]. Thus, the associated replicator dynamics [8] has a unique interior fixed point for $\gamma < rc - c$. This needs to be solved numerically for an arbitrary $k > 1$, but for $k = n - 1$ we reach the expression $x = \gamma/(c(n-1)(r-1) - \gamma(n-2))$. This fixed point is stable, because supporters of the pool can invade a monomorphous population of opportunists (as they sometimes benefit from inducing cooperation) and opportunists can also invade a monomorphous population of institution supporters (as they save the cost γ). The probability that a pro-social pool is successfully implemented is given by $\sum_{j=k}^{n} \binom{n}{j} x^{n-j}(1-x)^j$, where x is obtained from solving Eq 6.

D.1 Pairwise coexistences for $k = 2$ that cannot be invaded

For $k = 2$, there is again a coexistence between $WDC \circ C$ and $NDC \circ \circ$. Even for $k = 2$, this coexistence cannot be invaded by other strategies for our default parameters ($n = 5$, $r = 3$, $c = 1$, $\gamma = 0.7$, $\beta = 1.5$):

- **Invasion of other supporters of pro-social pools:** If $WDC \circ C$ players would cooperate in the absence of sanctions, they would be exploited, reducing their payoff.
- **Invasion of other players who do not support any institution:** If $NDC \circ \circ$ players would cooperate in the absence of sanctions, they would be exploited. If $NDC \circ \circ$ players would defect in the presence of a sanctioning pool, they would suffer from punishment.
- **Invasion of supporters of anti-social pools:** We assume that invaders continue to defect in the absence and cooperate in the presence of the pro-social pool, i.e. we focus on $MDCDD$. This provides the scenario most attractive for the supporters of anti-social sanctions. If a player would start to support the anti-social pool, her payoff would be identical to the $NDC \circ \circ$ players as long as no sanctions are implemented (invasion is neutral until the anti-social pool is implemented). We then compare the payoff these players can obtain to the one of the residents in their equilibrium, which implies that the number of mutants remains small.

  If the mutants find themselves in groups with only the anti-social pool, they pay the cost γ, but do not induce any cooperation and thus have a disadvantage compared to the residents. If the mutants find themselves in groups with both institutions, they are punished in addition. Since in the pairwise coexistence they invade, no player is punished, the fine implies a disadvantage for the supporters of the anti-social institution.

Thus, the coexistence between $WDC \circ C$ and $NDC \circ \circ$ is stable to any invasion also for $k = 2$.

D.2 Pairwise coexistences for $k = 2$ that can be invaded

A pairwise analysis of strategies for our default parameters ($n = 5$, $r = 3$, $c = 1$, $\gamma = 0.7$, $\beta = 1.5$) and $k = 2$ reveals that in addition to $WDC \circ C$ and $NDC \circ \circ$, other stable coexistences between two strategies are possible:

1. Coexistences between supporters of the pro-social pool and those who support no pools
   - A coexistence between $WDC \circ C$ and $NCC \circ \circ$ can be invaded by $NDC \circ \circ$ strategies.
   - A coexistence between $WDC \circ C$ and $NCD \circ \circ$ can be invaded by $NDD \circ \circ$ strategies, displaying unconditional defection.
2. Coexistences between supporters of the anti-social pool and those who support no sanctioning pools
   - A coexistence between $MD \circ DD$ and $NC \circ C\circ$ can be invaded by $ND \circ C\circ$.
   - A coexistence between $MD \circ DD$ and $NC \circ D\circ$ can be invaded by $ND \circ D\circ$.
3. Coexistences between supporters of the anti-social pool and supporters of the pro-social pool. In this case, there is always at least one institution implemented successfully, leading to three possible scenarios: Each pool alone or an implementation of both pools at the same time
   - A coexistence between $M \circ CDD$ and $W \circ CCC$ can be invaded by $W \circ CDC$.
   - A coexistence between $M \circ DDD$ and $W \circ CDC$ can be invaded by $M \circ CDD$.
   - There are two possible stable coexistences between $M \circ CDD$ and $W \circ CDC$. If the abundance of $M \circ CDD$ is high, it is more likely that the anti-social pool is implemented. Then, $N \circ CDD$ can invade. If the abundance of $W \circ CDC$ is high, it is more likely that the pro-social pool is implemented. Then, $N \circ CDC$ can invade.

   Thus, all coexistences that emerge for $k = 2$ that appear to be stable in a pairwise analysis are unstable with respect to a third invader strategy. Therefore, the qualitative results do no not change when we switch from $k = 1$ to $k = 2$, see Fig. 2. However, as there is now selection on the first bit of the pool supporter strategies to defect in the absence of sanctions, we only have six possible coexistences (displaying exactly the same behavior as for $k = 1$) instead of twelve.

E Additional costs for the sanctioning pools

Can cooperation be maintained if the supporters of the pro-social pool have an additional disadvantage by paying the cost γ for the institution even when the pool is not implemented? For $k = 1$, this situation never occurs. Only for $k > 1$, the additional costs affect the results and leads to a Volunteers Dilemma [13, 14]. In this case, the payoff of supporters of the pro-social pool is

$$\pi_{ID} = -\gamma + (rc - c) \sum_{j=k-1}^{n-1} \binom{n-1}{j} (1-x)^j x^{n-1-j}, \tag{7}$$

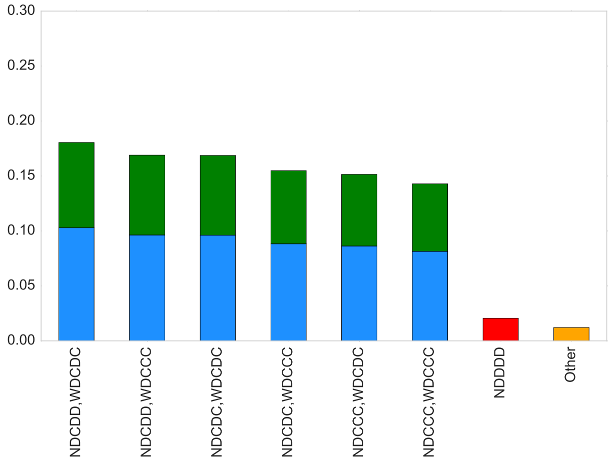

ESM Figure 2: **Evolutionary dynamics for the 20 consistent conditional strategies with $k = 2$.** More than 90% of the time the population is made up of a coexistence of conditional cooperators and sanctioning supporters. This indicates that the our results are robust to increasing the threshold of individuals required to establish punishment. The frequencies of cooperators and defectors when $k = 2$ indicate a similar pattern to that resulting from $k = 1$, i.e., a minority of sanctioning supporters coexists with a large number of conditional cooperators supporting no institution.

where the cost γ now always has to be payed. The payoff of opportunists remains unchanged. Now, $\pi_O = \pi_{ID}$ simplifies to

$$\frac{\gamma}{rc - c} = \binom{n-1}{k-1} x^{n-k} (1-x)^{k-1}. \tag{8}$$

The right hand side of this equation has a unique maximum at $x^* = \frac{n-k}{n-1}$ given by

$$\Gamma = \binom{n-1}{k-1} \left(\frac{k-1}{n-1}\right)^{k-1} \left(\frac{n-k}{n-1}\right)^{n-k}. \tag{9}$$

For $\Gamma > \frac{\gamma}{rc-c}$, the coexistence equilibrium vanishes and opportunists dominate over punishment supporters – no cooperation emerges. For $\Gamma < \frac{\gamma}{rc-c}$, there are two internal equilibria, one stable and one unstable. This implies that opportunists can invade and coexist with sanction supporters. But in contrast to the case of $k = 1$ and our usual assumption not implementing costs for a non-existing pool, now opportunism is stable and a substantial fraction of sanction supporters is necessary to reach the stable coexistence. This results in a complete breakdown of cooperation.

In this case — as in the case of unconditional strategies — the option not to take part in the game can provide an escape hatch out of mutual defection [15, 16]. If we add loners to our simulations, the system shows again some cooperation. Loners are necessary, as in the case of $k > 1$, opportunists can no longer invade defectors and an alternative path out of defection is necessary. However, for $k = 2$ and additional costs for the pools their success is very limited, see Fig. 3.

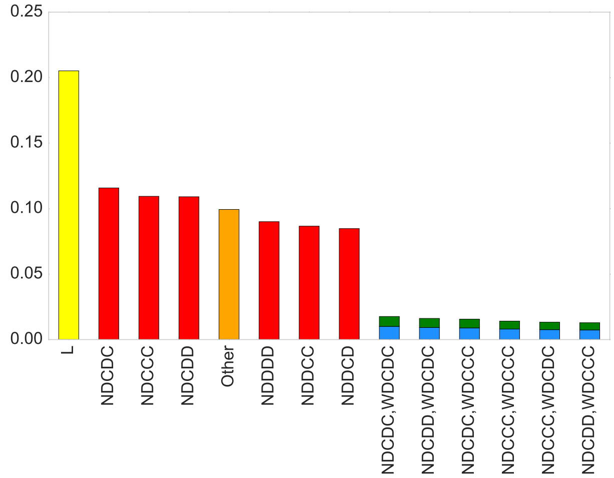

ESM Figure 3: **Additional costs for the institution in the case of $k = 2$.** When the pool also causes costs if its implementation fails, cooperation breaks down entirely in the absence of loners. Thus, we explore the role of loners in our simulations here and this explore the consistent conditional strategy set with 21 strategies. While loners provide a path out of defection, their success remains very limited here, as indicated by the low abundance of cooperative coexistences in this figure ($k = 2$, otherwise we use the standard parameter set).

F Additional costs on conditional strategies

Our model assumes that the cost for institutions to be visible is part of the funds that establish the institutions. However, it is also possible to assume that this cost is paid by those strategies that use conditional information. We can study this setup by considering a a cost $\delta > 0$, paid all individuals using strategies that discriminate. We will see that provided that the game is optional, the results remain valid in a wide range of parameters.

For the new cost it is important that δ is such that $0 < \sigma < (r-1)c - G - \delta$. That is, conditional cooperators in groups of cooperators still earn more than those that do not play the game.

The first thing to know is that the stable mixture that guarantees cooperation remains an equilibrium, provided $\delta < \beta - c$. First note that with the exception of $WCCCC$, any two strategies $W \circ C \circ C$ and $NDC \circ \circ$ will both pay the cost δ.

This coexistence cannot be invaded by any other single mutant:

- Individuals cooperating in the absence of an institution, $NCC \circ \circ$, would be exploited by the $NDC \circ \circ$ resident.
- Defectors in the presence of an institution, $NDD \circ \circ$, would be punished.
- Any player supporting the anti-social institution is outperformed by individuals in the stable coexistence: There, both players obtain $-\gamma + rc - c - \delta$. A single supporter of the anti-social institution would at most get $-\gamma + rc - \beta$. Therefore, supporters of an anti-social institution cannot invade provided $\delta < \beta - c$.

In addition for this equilibrium to be reached we require that the public goods game is optional. Our simulations show that the typical path into the stable mixture is via defection, which is neutral to $NDD\circ\circ$ (see Figure 3 in the main text). This path is no longer neutral if $NDD\circ\circ$ pays a cost δ, thus, getting into the mixture requires the loner strategy (see Figure 1 in this supplement).

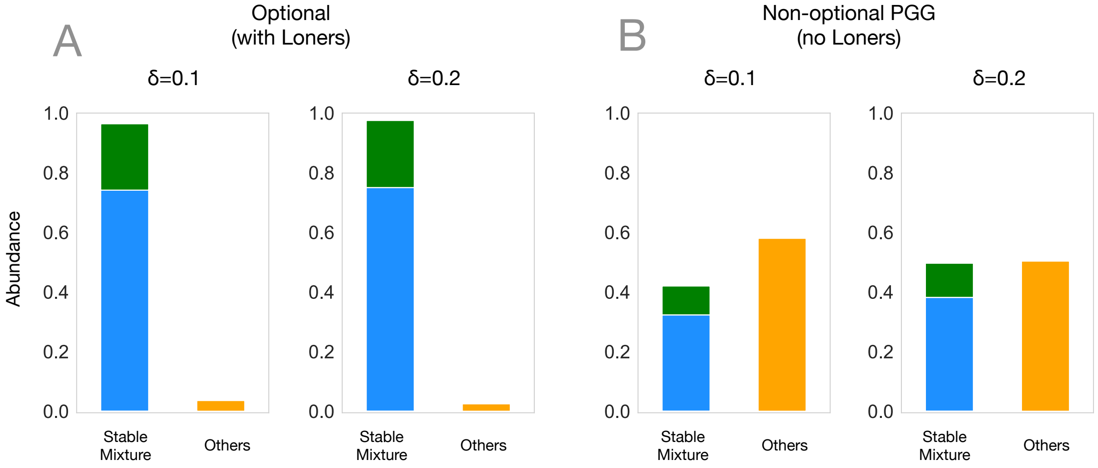

ESM Figure 4: **Additional costs for the conditional strategies.** The stable mixture is prevalent, provided the public goods game is optional (panel A), but is less prominent when the public goods game is mandatory (panel B). We show results for $\delta = 0.1$ and $\delta = 0.2$, other parameters as before, and averaging as before.

Figure 4 shows how simulations align with this prediction. A cost in the provided range leads to qualitatively similar results provided the public goods game is optional.

G Computational methods

The simulations reported follow a standard agent-based simulation of a Moran process. Fitness inside groups is deterministic and can be obtained using an algorithm that given a group composition computes the size of the pro-social and anti-social pool as well as the number of PGG contributors.

Given the payoff inside groups we can use a multivariate hypergeometric distribution in order to estimate the probability of each possible group composition. This is efficient when the number of types is small. When the population is composed of three or more types, we resort to Montecarlo simulations to estimate group sampling probabilities out of 1000 samples. However, the payoff computation is exact for the majority of simulation runs, since a small mutation rate guarantees that the population is at most dimorphic for most of the time.

The process that estimates the frequency of mixtures works in the following fashion. A file reporting a complete simulation run is post-processed as follows. At each reported time step, we remove strategies that are below a given noise threshold and compute the support of the population without noise. We then count how many times each support

appears as a fraction of all the time-steps in the simulation. Supports of size one correspond to monomorphous populations. Each support of size two corresponds to a potential stable mixture between two strategies. Supports of size three are reported, but their abundance is negligible given that mutation rates are small. The code from the simulations is available online. Payoff inside groups is determined with the following algorithm.

```
def payoff_against_group(focal_index, group_of_others, list_of_strategies):

    # create a copy of the group including the focal individual
    group_composition = copy(group_of_others)
    group_composition[focal_index] += 1

    # is the focal player a loner
    if list_of_strategies[focal_index] == 'L':
        return sigma

    # compute effective group size
    effective_group_size = len(group_composition) - number_of_loners
    if effective_group_size <= 1:
        # there is no game
        return sigma

    # determine the sizes of pools
    prosocial_pool_size = 0
    antisocial_pool_size = 0
    is_focal_a_prosocial_supporter = False
    is_focal_an_antisocial_supporter = False

    for i in range(0, len(group_composition)):
        if group_composition[i] > 0:
            if is_i_prosocial_supporter(i):
                prosocial_pool_size = prosocial_pool_size + group_composition[i]
                if i == focal_index:
                    is_focal_a_prosocial_supporter = True
            if is_i_antisocial_supporter(i):
                antisocial_pool_size = antisocial_pool_size + group_composition[i]
                if i == focal_index:
                    is_focal_an_antisocial_supporter = True

    # check if the threshold was reached
    if prosocial_pool_size < k:
        prosocial_pool_size = 0
    if antisocial_pool_size < k:
        antisocial_pool_size = 0

    # determine size of the PGG
    pg_pool_size = 0
    focal_cooperated = False
    for i in range(0, len(group_composition)):
        if group_composition[i] > 0:
            if do_i_cooperate_in_group(i, prosocial_pool_size, antisocial_pool_size):
                pg_pool_size = pg_pool_size + group_composition[i]
                if focal_index == i:
                    focal_cooperated = True

    # Given pool sizes and PGG size compute costs and benefits

    #Compute benefit
    if focal_cooperated:
        benefit = r * c * ((pg_pool_size - 1) / (effective_group_size - 1))
    else:
        benefit = r * c * (pg_pool_size / (effective_group_size - 1))

    # Compute fines
    fines = 0.0
    if not focal_cooperated:
        # prosocial institution punishes
        fines = fines +  beta * prosocial_pool_size
    else:
        # antisocial institution punishes
        fines = fines + beta * antisocial_pool_size

    # Compute insitutional cost
    institutional_cost = 0
    if is_focal_a_prosocial_supporter and prosocial_pool_size != 0:
        institutional_cost = institutional_cost + gamma
    if is_focal_an_antisocial_supporter and antisocial_pool_size != 0:
            institutional_cost =  institutional_cost + gamma
    # cost of cooperation
    if focal_cooperated:
        return benefit - fines - institutional_cost -  c
    else:
        return benefit - fines - institutional_cost
```

References

[1] Nowak MA, Sasaki A, Taylor C, Fudenberg D. 2004 Emergence of cooperation and evolutionary stability in finite populations. *Nature* **428**, 646–650.

[2] Traulsen A, Shoresh N, Nowak MA. 2008 Analytical results for individual and group selection of any intensity.. *Bulletin of Mathematical Biology* **70**, 1410–1424.

[3] Fudenberg D, Imhof LA. 2006 Imitation processes with small mutations. *Journal of Economic Theory* **131**, 251–262.

[4] Wu B, Gokhale CS, Wang L, Traulsen A. 2012 How small are small mutation rates?. *Journal of Mathematical Biology* **64**, 803–827.

[5] Antal T, Traulsen A, Ohtsuki H, Tarnita CE, Nowak MA. 2009 Mutation-selection equilibrium in games with multiple strategies.. *Journal of Theoretical Biology* **258**, 614–622.

[6] Traulsen A, Hauert C, De Silva H, Nowak MA, Sigmund K. 2009 Exploration dynamics in evolutionary games. *Proceedings of the National Academy of Sciences USA* **106**, 709–712.

[7] Wu B, García J, Hauert C, Traulsen A. 2013 Extrapolating weak selection in evolutionary games. *PLoS Computational Biology* **9**, e1003381.

[8] Hofbauer J, Sigmund K. 1998 *Evolutionary Games and Population Dynamics*. Cambridge, UK: Cambridge University Press.

[9] Diekmann A. 1985 Volunteer's Dilemma. *Journal of Conflict Resolution* **29**, 605–610.

[10] Weesie J, Franzen A. 1998 Cost sharing in a Volunteer's Dilemma. *Journal of Conflict Resolution* **42**, 600–618.

[11] Schoenmakers S, Hilbe C, Blasius B, Traulsen A. 2014 Sanctions as honest signals - The evolution of pool punishment by public sanctioning institutions. *Journal of Theoretical Biology* **356**, 36–46.

[12] Peña J, Lehmann L, Nöldeke G. 2014 Gains from switching and evolutionary stability in multi-player matrix games. *Journal of Theoretical Biology* **346**, 23–33.

[13] Taylor M, Ward H. 1982 Chickens, whales, and lumpy goods: alternative models of public-goods provision.. *Political Studies* **30**, 350–370.

[14] Bach LA, Helvik T, Christiansen FB. 2006 The evolution of N-player cooperation - threshold games and ESS bifurcations. *Journal of Theoretical Biology* **238**, 426–434.

[15] Hauert C, De Monte S, Hofbauer J, Sigmund K. 2002 Volunteering as Red Queen mechanism for cooperation in public goods games.. *Science* **296**, 1129–1132.

[16] Hauert C, Traulsen A, Brandt H, Nowak MA, Sigmund K. 2007 Via freedom to coercion: the emergence of costly punishment. *Science* **316**, 1905–1907.
